# Structure and metal-binding properties of PA4063, a novel player in periplasmic zinc trafficking by *Pseudomonas aeruginosa*

**DOI:** 10.1107/S2059798321009608

**Published:** 2021-10-20

**Authors:** Annarita Fiorillo, Andrea Battistoni, Serena Ammendola, Valerio Secli, Serena Rinaldo, Francesca Cutruzzolà, Nicola Demitri, Andrea Ilari

**Affiliations:** a Department of Biochemical Sciences, Sapienza University of Rome, Pizzale Aldo Moro 5, 00185 Rome, Italy; b Institute of Molecular Biology and Pathology (IBPM), National Research Council of Italy (CNR), Pizzale Aldo Moro 5, Rome, Italy; c Department of Biology, University of Tor Vegata, Via delle Ricerca Scientifica 1, Rome, Italy; d Laboratory Affiliated To Istituto Pasteur Italia – Fondazione Cenci Bolognetti, Rome, Italy; e Elettra-Sincrotrone Trieste S.C.p.A., S.S. 14 km 163.5 in Area Science Park, Basovizza, 34149 Trieste, Italy

**Keywords:** PA4063, periplasmic zinc trafficking, *Pseudomonas aeruginosa*, ferredoxin-like fold

## Abstract

The capability to obtain essential nutrients in hostile environments is a critical skill for pathogens. Under zinc-deficient conditions, *Pseudomonas aeruginosa* expresses a pool of metal homeostasis control systems that is complex compared with other Gram-negative bacteria and has only been partially characterized. Here, the structure and zinc-binding properties of the protein PA4063, the first component of the PA4063–PA4066 operon, are described. PA4063 has no homologs in other organisms and is characterized by the presence of two histidine-rich sequences. ITC titration detected two zinc-binding sites with micromolar affinity. Crystallographic characterization, performed both with and without zinc, revealed an α/β-sandwich structure that can be classified as a noncanonical ferredoxin-like fold since it differs in size and topology. The histidine-rich stretches located at the N-terminus and between β3 and β4 are disordered in the apo structure, but a few residues become structured in the presence of zinc, contributing to coordination in one of the two sites. The ability to bind two zinc ions at relatively low affinity, the absence of catalytic cavities and the presence of two histidine-rich loops are properties and structural features which suggest that PA4063 might play a role as a periplasmic zinc chaperone or as a concentration sensor useful for optimizing the response of the pathogen to zinc deficiency.

## Abstract 2

The structural and zinc-binding properties of PA4063, one of the periplasmic proteins expressed by *Pseudomonas aeruginosa* under zinc-deficient conditions, are presented. PA4063 has a noncanonical ferredoxin-like fold. Two zinc-binding sites with micromolar affinity are exposed and are located on the same face of the structure. Two histidine-rich loops, which are disordered in the apoprotein, contribute to zinc coordination in one of the sites. These findings strongly suggest a role for PA4063 in zinc trafficking, possibly acting as a metal chaperone or as a regulator of a transport system.

## 1. Introduction   

Zinc is an essential element for all known living organisms, being an indispensable structural or functional cofactor in many different proteins. Indeed, it is estimated that zinc is used as a cofactor by more than 5% of bacterial proteins (Andreini *et al.*, 2006 ▸).

The acquisition of zinc, as well as other metals, is one of the many battlefields in the host–pathogen fight. Indeed, so-called nutritional immunity is a strategy implemented by the host to oppose infection, consisting of lowering the concentration of free metals to affect microbial viability. On the other hand, the opposite strategy, in which the host exploits the toxicity of high metal concentrations to kill the parasite, has also been reported (Hood & Skaar, 2012 ▸). Therefore, parasites need very efficient systems to regulate the trafficking of metals to counteract host defence and survive in inhospitable environments in general.

The outer membrane of Gram-negative bacteria is thought to be permeable to low-molecular-weight hydrophilic molecules and to small ions (Delcour, 2009 ▸). Thus, in a zinc-rich environment, zinc ions may passively diffuse from the extracellular space into the periplasm, where the metal is bound by specific proteins which mediate its transport across the inner membrane to the cytoplasm (Battistoni *et al.*, 2017 ▸).

The intracellular supply of zinc is primarily ensured by low-affinity importers such as ZupT, an integral inner membrane protein that belongs to the ZIP (ZRT, IRT-like protein) family and has the capability to mediate the import of a broad range of divalent metals, with a preference for zinc ions (Grass *et al.*, 2002 ▸). When the zinc concentration falls below a critical value, other proteins are expressed to increase the ability of the bacteria to bind the metal and to adapt to conditions of zinc paucity (Cerasi *et al.*, 2013 ▸). The gene expression of these proteins is controlled by Zur (zinc-uptake regulator), a transcription factor which, under zinc-replete conditions, binds the metal and undergoes a conformational change that allows it to bind to the promoter region of controlled genes, preventing their expression. Conversely, when the intracellular zinc concentration becomes low, Zur releases zinc and detaches from the promoter, allowing gene transcription (Outten *et al.*, 2001 ▸).

The pool of proteins regulated by Zur is highly variable from species to species. Whereas in some microbial species the Zur regulon comprises just a small number of genes, in other species this number increases significantly and includes genes whose functions have still to be clarified. It is possible to hypothesize that this difference in the complexity of the response to zinc deficiency is related to the ability to colonize environments where zinc paucity is particularly acute or to the fact that in these microorganisms this metal performs more numerous or critical functions than in other species. One of the microorganisms that possesses a particularly complex and still poorly investigated Zur regulon is *Pseudomonas aeruginosa*, a bacterium that is responsible for numerous opportunistic infections in humans.

A key element in most Gram-negative bacteria is ZnuABC, a high-affinity zinc importer and a member of the wide and ubiquitous ABC (ATP-binding cassette) superfamily. Like all bacterial ABC transporters, ZnuABC consists of three components: a periplasmic high-affinity solute-binding protein (ZnuA), a permease (ZnuB) and an ATPase (ZnuC) that furnishes the energy for ion translocation to the cytosol. The X-ray structures of ZnuA, first solved for *Synechocystis* 6803 (Banerjee *et al.*, 2003 ▸) and subsequently for *Escherichia coli* and *Salmonella enterica* (Li & Jogl, 2007 ▸; Ilari *et al.*, 2011 ▸), show that it displays the typical Venus fly-trap fold of periplasmic solute-binding proteins (PBPs), in which a high-affinity zinc-binding site is located at the interface of two globular (α/β)_4_ domains. At the entrance to the site, a distinctive characteristic of ZnuA is located: a histidine-rich (His-rich) loop that plays a significant, although still debated, role in the zinc-management process (Blindauer, 2015 ▸). Some bacteria also encode ZinT, a Zur-controlled periplasmic protein that acts as an auxiliary component of the ZnuABC system and that contributes to zinc recruitment in the periplasm by interacting with ZnuA (Petrarca *et al.*, 2010 ▸; Ilari *et al.*, 2014 ▸).

*P. aeruginosa* expresses a zinc-regulated ZnuABC zinc importer but, unlike in other bacteria, it is not essential for proliferation in zinc-poor media. This is possible because this microbe is endowed with other proven or putative metal-uptake systems regulated by Zur, such as ZrmABCD, HmtA, PA2911–PA2914 and PA4063–PA4066 (Pederick *et al.*, 2015 ▸), which are briefly presented below. The most studied among these is ZrmABCD, which is involved in the synthesis and management of pseudopaline, a low-molecular-weight chelating agent that mediates the import of zinc in a siderophore-like manner (Mastropasqua *et al.*, 2017 ▸; Lhospice *et al.*, 2017 ▸; McFarlane & Lamb, 2017 ▸; Hermansen *et al.*, 2018 ▸; Gomez *et al.*, 2021 ▸). The importance of this system is shown by the fact that the inactivation of both ZnuABC and ZrmABCD reduces the ability of *P. aeruginosa* to proliferate in zinc-poor environments and drastically reduces its ability to cause disease in mice, possibly due to a significant reduction of the expression of several virulence features such as biofilm formation, swimming ability, extracellular protease production and siderophore release (D’Orazio *et al.*, 2015 ▸; Mastropasqua *et al.*, 2017 ▸, 2018 ▸).

HmtA (heavy-metal transporter A), also known as Q9I147, is a P-type ATPase family member. Its contribution to metal import in *P. aeruginosa* has never been clearly assessed, but studies carried out in *E. coli* have shown that it is able to pump zinc and other metals such as copper into the cell (Lewinson *et al.*, 2009 ▸).

The PA2911–PA2914 operon is poorly characterized. By sequence analysis, it encodes a metal-transport system homologous to iron ABC permeases (PA2912–PA2914) and a putative TonB-dependent receptor (PA2911) (Pederick *et al.*, 2015 ▸), and overall it has been proposed to mediate the transport of chelated zinc from the extracellular environment to the cytoplasm.

PA4063–PA4066 is the most elusive among the operons presently associated with zinc deficiency in *P. aeruginosa*. It comprises two genes annotated as members of an ABC transporter (PA4064 and PA4065), while the other two genes encode periplasmic proteins with less apparent function. To start a functional characterization of this operon, we have focused on its first component, PA4063. This gene encodes a protein with poor similarity to any other known proteins but, like ZnuA, it contains stretches rich in histidines, indicating a probable direct involvement in the management of zinc. In this paper, we describe the X-ray crystal structures of free and zinc-bound PA4063 in two different crystal forms, revealing a peculiar ferredoxin-like fold and disclosing the details of metal binding, which are suggestive of a role as a metallochaperone or zinc sensor.

## 2. Materials and methods   

### 2.1. Cloning of PA4063   

The sequence encoding the mature PA4063 protein (without the signal sequence for periplasmic export) was amplified using the chromosomal DNA from *P. aeruginosa* PAO1 as a template and the oligonucleotides PA4063_5 (CACCATGGCCCATGACGACCACGACCATGA) and PA4063_6 (GTGAATTCTACAGCTTCAGCTCGGCG), bearing NcoI and EcoRI cloning sites at the 5′-and 3′-termini, respectively. PCR was carried out using an Expand High Fidelity PCR System (Sigma–Aldrich). The amplified DNA fragment was then digested with NcoI and EcoRI, run on a preparative agarose gel (TAE) and purified using a Zymoclean Gel DNA Recovery Kit (Zymo Research). This fragment was subsequently inserted into the NcoI and EcoRI restriction sites of the expression vector pET-26b(+), generating plasmid pET-26b(+)PA4063. In this vector, the sequence encoding mature PA4063 is fused to the *Erwinia carotovora pelB* signal sequence, thus allowing periplasmic localization of the expressed protein. The sequence of the insert was subsequently confirmed by nucleotide sequencing. The vector was inserted into *E. coli* BL21 (DE3) cells for protein expression.

### 2.2. PA4063 expression and purification   

*E. coli* BL21 (DE3) cells bearing pET-26b(+)PA4063 were inoculated into 4 l lysogeny broth at 37°C and expression of PA4063 was induced in mid-log phase cultures by the addition of 1 m*M* isopropyl β-d-1-thiogalactopyranoside (IPTG). After 24 h of growth, the cells were harvested by centrifugation and resuspended in 20% sucrose, 30 m*M* Tris–HCl pH 8.0, 1 m*M* EDTA. Afterwards, lysozyme was added to a concentration of 1 mg ml^−1^. After 10 min incubation on ice, the bacteria were centrifugated at 12 000*g* and the supernatant, containing the periplasmic proteins, was collected and extensively dialyzed against 50 m*M* NaH_2_PO_4_, 250 m*M* NaCl pH 7.8. Subsequently, the protein extract was loaded onto an Ni–NTA column (20 ml bed volume) pre-equilibrated with the same buffer. Proteins were fractionated according to their affinity for immobilized metal ions using a 0–250 m*M* linear gradient of imidazole. Most of the proteins eluted at imidazole concentrations below 60 m*M*, whereas PA4063 eluted as a pure protein at imidazole concentrations above 100 m*M*. Fractions containing PA4063 were collected, concentrated to a small volume and then extensively dialyzed against 20 m*M* HEPES, 100 m*M* NaCl pH 7.0. Protein concentration was determined by the method of Lowry.

Apo PA4063 was obtained by dialyzing the protein for 24 h against 50 m*M* sodium acetate, 2 m*M* EDTA pH 5.5. EDTA was then removed by dialysis against a buffer consisting of 50 m*M* sodium acetate, 100 m*M* NaCl pH 5 and the protein was finally dialyzed in 20 m*M* HEPES, 100 m*M* NaCl pH 7.0. To minimize metal contamination, all of these dialyses were carried out in buffers prepared with Chelex-treated water and in glassware pre-treated with nitric acid.

### 2.3. UV–Vis spectrophotometric analyses   

Since zinc is spectrophotometrically silent, we analysed the ability of the protein to bind cobalt, a metal that has the ability to easily substitute for zinc in metal-binding proteins *in vitro* or *in vivo* (Ammendola *et al.*, 2020 ▸). Experiments were carried out with apo PA4063 at a concentration of 670 µ*M*. Spectra were recorded in the UV–Vis region following the addition of progressively increasing amounts of metal corresponding to 0.5 molar equivalents of the protein. Each spectrum was recorded immediately after the metal was added, subsequently evaluating any variations over time due to possible slow conformational changes induced by the metal. To gain insight into the ability of PA4063 to bind zinc, we analyzed the effect of zinc addition on the spectrum of the cobalt-bound protein. For this purpose, we carried out a gradual addition of zinc sulfate in aliquots of 0.5 zinc equivalents, as previously performed with cobalt.

### 2.4. Isothermal titration calorimetry (ITC)   

ITC experiments were carried out using an iTC200 microcalorimeter (MicroCal, Malvern). 1.2 µl aliquots of metal solution (0.32 m*M* zinc acetate or cobalt nitrate) were injected into 11 µ*M* apo PA4063 solution at 25°C. The experiments were carried out in the dialysis buffer used to prepare the apoprotein (20 m*M* HEPES pH 7.0, 100 m*M* NaCl) diluted with the percentage of water used to prepare the metal solutions. 0.32 m*M* metal solutions were prepared from water-dissolved 2 m*M* stock solutions. Data were fitted using the one-binding-site model of the MicroCal PEAQ-ITC Analysis Software (Malvern) version of *Origin*. The heat of binding (Δ*H*), the stoichiometry (*n*) and the dissociation constant (*K*_d_) were then calculated from plots of the heat evolved per mole of ligand injected versus the molar ratio of ligand to protein using the software provided by the vendor. The data are the mean ± standard deviation of three or two experiments for zinc or cobalt, respectively. Optimization of the proper protein:ligand concentration ratio used to obtain the thermodynamic signature reported in this manuscript was carried out by assaying different protein concentrations (5.9, 7, 9.6 and 11 µ*M* with zinc ligand; data not shown).

### 2.5. Crystallization   

Automated crystallization screening (Crystal Phoenix, Art Robbins Instruments) was performed at 294 K in a sitting-drop vapour-diffusion setup using a 1:1 ratio of protein and reservoir solutions (0.6 µl drop volume, 70 µl reservoir). The protein solution consisted of 10 g l^−1^ PA4063, 20 m*M* HEPES pH 7.5, 100 m*M* NaCl. Screening was performed with and without 5 m*M* zinc acetate added to the protein solution (zinc:protein ratio of about 10:1). Two crystallization conditions (A and B) were identified in the absence of zinc. Condition A, consisting of 2 *M* ammonium sulfate and 2 *M* NaCl, produced a crystal with tetragonal symmetry (space group *P*4_1_2_1_2) that diffracted to 1.95 Å resolution but could not be easily reproduced. Condition B, consisting of 2.7–3 *M* ammonium sulfate and 1% 2-methyl-2,4-pentanediol (MPD), produced poorly diffracting rod-shaped crystals with hexagonal symmetry (space group *P*6_5_) that were further optimized by hand and by microseeding, growing up to 0.5 mm in length, which reached a maximum resolution of 2.7 Å.

Since crystallization did not occur in the presence of zinc, crystal soaking was performed to identify zinc-binding sites. Crystals from both conditions A and B were soaked in 5 m*M* zinc acetate (zinc:protein ratio of around 10:1) for 1 h.

### 2.6. Data collection and structure solution   

PA4063 has no sequence similarity to any protein present in the PDB; therefore, phasing was accomplished by single-wavelength anomalous dispersion (SAD) experiments. The presence of a single methionine in the sequence led us to prefer heavy-atom derivatization over selenomethionine substitution. A native diffraction data set was collected from a tetragonal crystal to a resolution of 1.95 Å using a wavelength of 1 Å. However, the better-diffracting tetragonal crystals were not easily reproducible; thus, heavy-atom derivatization was only attempted on the hexagonal crystals. Crystals were soaked in 5 m*M* KAu(CN)_2_ for 1 h followed by back-soaking in mother liquor (three quick steps in 2 µl drops, the last with an additional 20% glycerol). The best SAD data set was collected at the gold absorption peak (λ = 1.000 Å) to a resolution of 2.7 Å.

A zinc-soaked hexagonal crystal was used for X-ray diffraction data collection at the wavelength corresponding to the zinc absorption peak (λ = 1.271 Å) to 3.3 Å resolution.

Three diffraction data sets were collected on the XRD2 beamline at the Elettra synchrotron-radiation source, Trieste, Italy using a Dectris PILATUS 6M detector at 100 K (Lausi *et al.*, 2015 ▸).

An additional data set was collected to 2.82 Å resolution from a zinc-soaked tetragonal crystal at a wavelength of 0.967 Å. This last data set was collected using a Dectris EIGER2 X 4M detector on beamline ID30B at the ESRF synchrotron-radiation source, Grenoble, France.

The data sets were processed and scaled with *XDS* (Kabsch, 2010 ▸) and *AIMLESS* (Evans & Murshudov, 2013 ▸).

SAD phasing was accomplished by exploiting the gold anomalous signal with *SHELXC*/*D*/*E* (Sheldrick, 2010 ▸). A significant anomalous signal was detected to 3.4 Å resolution (*i.e.* where CC_anom_ falls below a 0.15 threshold). *SHELXD* located two gold sites with occupancies of 1 and 0.33, and subsequent phasing and density modification with *SHELXE* allowed 147 residues to be autotraced in space group *P*6_5_ (the final model has an estimated mean FOM of 0.521 and a pseudo-free CC of 58.80%). Model building of one monomer in the asymmetric unit was performed either automatically using *Buccaneer* (Cowtan, 2006 ▸) or manually with *Coot* (Emsley *et al.*, 2010 ▸). The traced monomer was used to perform molecular replacement with *MOLREP* (Vagin & Teplyakov, 2010 ▸) to find the locations of the other two monomers in the trimeric asymmetric unit. The structure was refined with *REFMAC*5 (Murshudov *et al.*, 2011 ▸). Only the gold ion corresponding to the stronger peak was included in the final model (the final occupancy refined to 0.72) since the putative second site, located in a completely hydrophobic small pocket, appeared to be unconvincing. The PA4063 structure at 2.7 Å resolution obtained from the hexagonal crystal was then used to solve the higher resolution structure of PA4063 from the tetragonal crystal (resolution of 1.95 Å) by molecular replacement.

Structural coordinates have been deposited in the Protein Data Bank with accession codes 7ahw (apo tetragonal crystal), 7aly (gold derivative, hexagonal crystal), 7bgo (zinc-bound, hexagonal crystal) and 7amx (zinc-bound, tetragonal crystal). Data-collection and refinement statistics are summarized in Table 1 ▸.

## 3. Results   

### 3.1. Metal-binding properties of PA4063   

**Figure 1:**
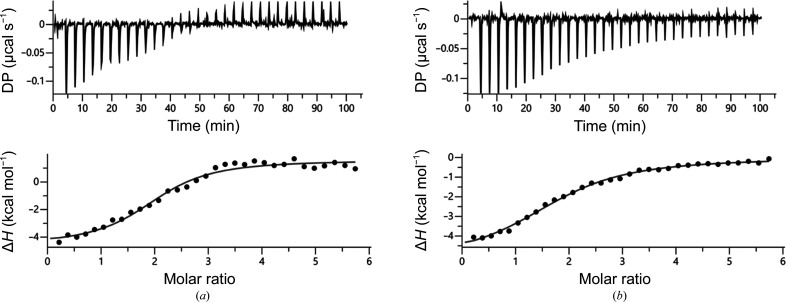
Binding of selected metals to apo PA4043. Analysis by ITC measurements was performed by titrating 11 µ*M* PA4063 with 320 µ*M* zinc acetate (*a*) or cobalt nitrate (*b*) solution in 84% dialysis buffer (150 m*M* NaCl, 20 m*M* Tris pH 7.2). The upper panels show the raw ITC data, while the lower panels show the integrated energy values normalized for injected protein. Binding isotherms were fitted using a single-binding-site model.

Preliminary UV–Vis spectrophotometric analysis revealed that PA4063 can bind either zinc or cobalt, showing a higher affinity for zinc (Supplementary Fig. S1). To obtain quantitative information on the number of metal-binding sites and on the stability of the protein–metal interaction, ITC experiments were run on the apoprotein. The titration profiles with zinc or cobalt ions are shown in Fig. 1 ▸. The dissociation constant (*K*_d_) and all of the additional thermodynamic parameters obtained by fitting the enthalpy curves are reported in Table 2 ▸ and Supplementary Fig. S2, respectively.

As expected for specific binding, integration of the titration peaks of apo PA4063 produced a sigmoidal enthalpy curve for each metal (Fig. 1 ▸, black markers) and a binding stoichiometry of ∼2. Under these experimental conditions, data fitted with the single-site-binding equation indicate that the two binding sites on the protein are equivalent, displaying the same affinity and the same mechanism of binding for each metal. In fact, the thermodynamic signature (*i.e.* profiling of the thermodynamic parameters; Supplementary Fig. S2) includes favourable enthalpic and entropic factors in both cases, thus suggesting that both binding events are mainly guided by novel inter­actions and dehydration processes that take place upon metal coordination, rather than by conformational change(s) of the protein. On the other hand, comparison of the *K*_d_ for the two metals indicates that the affinity of apo PA4063 for zinc is twofold higher than that for cobalt.

### 3.2. Apo PA4063 structure   

PA4063 crystallized in two different forms corresponding to space groups *P*4_1_2_1_2 and *P*6_5_. The asymmetric unit of the hexagonal crystal comprises three protein molecules, but the interactions are so limited that they can be considered as simple packing contacts between monomers as in the case of the tetragonal crystal form. This observation is in line with size-exclusion chromatography, which shows that the protein is monomeric in solution regardless of the presence of zinc (Supplementary Fig. S3).

Despite the differences in crystal packing with regard to both lattice and asymmetric unit composition, the conformation of the protein in the two crystal forms is almost identical (C^α^ r.m.s.d. in the range 0.49–0.74 Å). Thus, since the tetragonal crystal diffracted to higher resolution (1.95 Å versus 2.7 Å), the structural analysis presented here focuses on the latter model.

**Figure 2:**
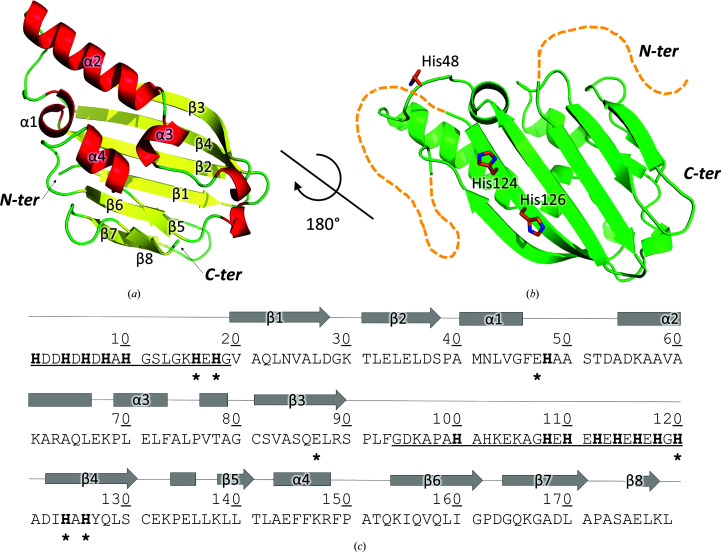
Structure of apo PA4063. Two views of the structure showing the layers of the α/β sandwich. (*a*) The α-helical side; secondary-structure elements are indicated. (*b*) The β-sheet side: the unstructured His-rich stretches, underlined in the sequence, are represented as orange dashes and histidines located in the folded region are represented as orange sticks. (*c*) Residues involved in zinc coordination are marked with asterisks, histidines are in bold and unstructured regions are underlined.

The structure is monomeric, with an α/β-sandwich architecture resembling the ferredoxin fold, although relevant differences can be detected. Indeed, PA4063 comprises a six-stranded antiparallel β-sheet made up of three β-hairpins (β3β4–β2β1–β6β7) interrupted in sequence by two main α-helices, four short helical turns and a small two-stranded β-sheet (β5–β8), collectively constituting the second layer of the ‘sandwich’ (Fig. 2 ▸). The secondary structure of PA4063 can then be represented as ββ(α)α(αα)ββ(αβ)αββ(βα), where minor elements are shown in parentheses. A disulfide bridge connects Cys81 on β3 and Cys131 on β4.

Comparison with the canonical ferredoxin-like fold, with a signature βαββαβ secondary structure, reveals that PA4063 differs in both composition and arrangement of β elements. In fact, even when neglecting minor elements, PA4063 has two more β-strands at the ends of the sequence that are inserted in the sheet to form β-hairpins, implying that the strands are coupled both in sequence and structure, while in ferredoxin the sheet is organized such that only two of the strands that are consecutive in sequence are contiguous in the 3D structure (see Supplementary Fig. S4 for a topology diagram).

His-rich sequences of 19 and 24 amino acids, located at the N-terminus and between β3 and β4, respectively, are disordered and are not visible in the crystal structure, as usually observed in other proteins containing this motif (Blindauer, 2015 ▸; Ilari *et al.*, 2011 ▸). It is worth noting that both His-rich fragments face the same side of the structure, corresponding to the region where the two zinc ions bind (see below). Only three histidines belong to the structured portion, two of which (His124 and His126) are located on β4 and the third of which (His48) is on the loop between β2 and the following helix (Fig. 2 ▸).

### 3.3. Zinc-bound PA4063 structure   

The structure of PA4063 in complex with zinc was solved by soaking crystals in zinc acetate solution since crystallization did not occur in presence of zinc. Soaking experiments were first performed with hexagonal crystals, despite their lower X-ray diffraction power, due to the poor reproducibility of the tetragonal crystals, leading to data collection to 3.3 Å resolution.

**Figure 3:**
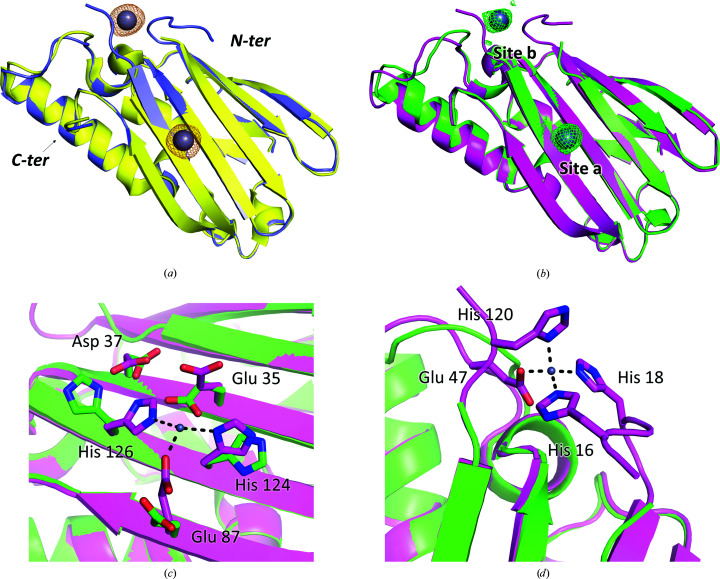
Zinc-bound PA4063 structure. (*a*) Superimposition of apo (yellow) and zinc-bound (blue) PA4063 from the hexagonal crystal form. The anomalous difference density map (orange mesh), contoured at 5σ, unequivocally locates two zinc-binding sites. (*b*) Superimposition of apo (green) and zinc-bound (magenta) PA4063 from the better-diffracting tetragonal crystal form. The difference map (*mF*_o_ − *DF*_c_, green mesh), set to 3σ, confirms the position of the zinc ions. (*c*) and (*d*) show the details of zinc coordination in sites **a** and **b**, respectively.

Diffraction data were collected close to the absorption edge of zinc (λ = 1.27 Å) in order to unambiguously locate the metal. Two strong anomalous peaks were observed for each monomer (>9σ), revealing two binding sites, according to the ITC experiments, indicated as site **a** and site **b** (Fig. 3 ▸ and Supplementary Fig. S5).

Later, a higher resolution (2.85 Å) data set was collected from a zinc-soaked tetragonal crystal, confirming the positions of the two zinc-binding sites and allowing a better determination of the complex geometry and coordination distances.

Site **a** is located on the flat surface of the β-sheet in correspondence with the histidine residues. The same mode of binding is detected in all three monomers of the hexagonal crystal: besides His124 and His126, the zinc ion binds to Glu87, likely as a monodentate ligand, and a water molecule, adopting a tetrahedral coordination geometry (Fig. 3 ▸ *c*). The water molecule is not visible in the tetragonal crystal, where zinc appears to be coordinated by only three ligands.

All distances between zinc and ligands are in the range 2–2.5 Å. Comparison with the apo structure shows that coordination requires rearrangement of the donor side chains, which move closer to the site, while Glu35 is displaced. The second shell of coordination comprises many acidic amino acids (Glu33, Glu35, Asp37 and Asp122) that facilitate zinc binding through a double mechanism. Indeed, the global negative charge could have the effect of attracting cations to the site and increasing the basicity of the histidines, thus favouring metal coordination.

Site **b** is about 20 Å from site **a**, on the edge of the β-sheet where the His-rich sequences are inserted. Notably, a few residues belonging to these sequences, which are disordered in apo PA4063, become structured and take part in zinc coordination, although some variability is present. As for site **a**, the coordination is tetrahedral and three donor groups are provided by one glutamate, Glu47, and two histidines, His16 and His18 (Fig. 3 ▸ *d*). Indeed, 5–6 additional residues (14/15–19) are visible at the N-terminus with respect to the apo structure. The fourth donor was modelled as a water molecule in monomer *A* of the hexagonal crystal, while in monomers *B* and *C*, as well as in the tetragonal crystal, the electron-density map indicates His120 to be the donor. Even in this case, the residue belongs to the His-rich stretch and only becomes structured upon zinc binding.

When evaluating the contribution of the His-rich sequences to the binding and the structural variation thus induced, it must be considered that the zinc-bound structure was obtained by soaking, thus the rearrangement could be influenced and limited by crystal packing. On the other hand, the fact that crystallization does not take place in the presence of zinc supports the hypothesis that the His-rich stretches participate in the binding but in a flexible and inhomogeneous way, thus interfering with the crystallization process.

### 3.4. Surface analysis   

**Figure 4:**
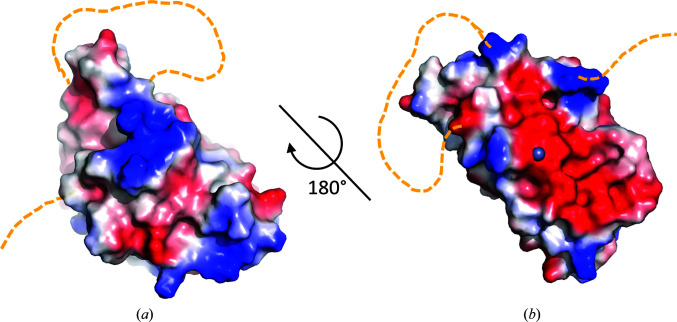
Surface analysis of PA4063. The picture shows two different views of the electrostatic surface potential of zinc-bound PA4063 generated with *PyMOL*. The protein orientation is the same as shown in Fig. 2 ▸: (*a*) α-­helical side, (*b*) β-sheet side. Disordered His-rich stretches are represented as orange dashed lines. In (*b*) the zinc ion in site **a** is represented as a grey sphere, while that in site **b** is buried and not visible.

To delve into the structural characterization, we analysed the shape and charge distribution of the surface of PA4063 (Fig. 4 ▸). The protein has no relevant cavities and this feature, together with the exposure of both zinc-binding sites to solvent and the relatively low metal affinity, indicates that a catalytic function is improbable. The electrostatic potential surface reveals an extended negative patch on the flat surface of the β-sheet, which is likely to contribute to metal binding to site **b**, while the α-helical face has a rather positive character.

A similar charge distribution is observed in Atx1, a ferredoxin-like copper chaperone present in eukaryotes that is responsible for metal transfer to the ATPase Ccc2. In the case of Atx1, the positive surface was indicated as critical for protein–protein interaction (Banci *et al.*, 2006 ▸), while no role was proposed for the negative surface, which does not correspond to a metal-binding site in this protein.

## 4. Discussion   

*P. aeruginosa* is an opportunistic pathogen that affects immunocompromised patients. It is known to be the leading cause of morbidity and mortality in cystic fibrosis patients and to be one of the primary causes of nosocomial infections. Due to its range of mechanisms for adaptation, survival and resistance to multiple classes of antibiotics, infections by *P. aeruginosa* strains can be life-threatening and it is emerging worldwide as a public health threat. Among the many factors contributing to the pathogenic potential of this microorganism is its ability to proliferate in environments that are very low in zinc or that contain high concentrations of the metal by taking advantage of several efficient Zn^2+^-uptake and Zn^2+^-efflux systems (Gonzalez *et al.*, 2019 ▸). When the concentration of zinc decreases, metal-import systems controlled by Zur are expressed and, among these, ZnuABC stands out as being very effective and nearly ubiquitous in Gram-negative bacterial species. However, unlike other bacteria such as *S. enterica* or *E. coli*, *P. aeruginosa* can proliferate in zinc-poor media even in the absence of ZnuABC due to its ability to express additional putative metal-transport systems, one of which is the still uncharacterized operon PA4063–PA4066.

Based on sequence analysis, PA4064 and PA4065 are likely to be part of an ABC transporter, as annotated in the UniProt database. Indeed, a search for proteins homologous to PA4064, performed with *BLAST* against the PDB, allowed the identification of ten proteins with a query cover greater than 90% and a sequence identity greater than 30%, all belonging to the ATP-binding component of ABC transporters. In particular, the nucleotide-binding domain of the macrolide export ATP-binding/permease protein MacB (PDB entry 5lj9; Crow *et al.*, 2017 ▸) displays 35.5% identity to PA4064. The same search for PA4065 yielded no homologous proteins with known structure; however, sequence analysis with *PredictProtein* (https://predictprotein.org/) suggests that PA4065 could be the permease component of an ABC transporter with four transmembrane helices. The crystal structure of PA4066 (PDB entry 4hsp) has been solved by the Joint Center for Structural Genomics, but neither the fold nor sequence analysis give any reasonable clue to its role. In fact, according to protein structure classification by both CATH and SCOP (http://www.cathdb.info/, https://scop.mrc-lmb.cam.ac.uk/) PA4066 is the only member of a family with unknown function that is apparently related to DNA-binding proteins, due to its similarity to the oligonucleotide-binding (OB) fold, although the periplasmic localization of the protein is in contrast to this activity. For PA4063, sequence analysis only reveals the presence of two long stretches rich in histidines (His-rich loops); no homology could be detected with any other characterized protein.

Here, we describe the structural characterization of the first component of the PA4063–PA4066 operon, with the aim of shedding light on its function. Our work defines some important characteristics of PA4063, which can be summarized as follows: (i) the protein has a noncanonical ferredoxin-like fold with no relevant cavities and the two His-rich loops are mostly disordered and are placed on the same side of the structure, (ii) ITC experiments reveal two zinc-binding sites with micromolar affinity, (iii) the zinc-binding sites are exposed and located on the same surface into which the His-rich loops are inserted and (iv) metal coordination involves one aspartate and two or three histidine residues and one of the sites requires a partial disorder-to-order transition of the two His-rich loops.

Given the absence of cavities and the relatively low affinity for zinc, catalytic activity can be excluded and, on the whole, the results support the hypothesis of a role for PA4063 in zinc transport and/or homeostasis.

The overall structure itself does not provide strong evidence about the function of PA4063, considering that the ferredoxin-like fold is found in a huge number of proteins and none of these displays the same topology as found in our target, as discussed in Section 3. Indeed, according to the SCOP classification, the ferredoxin-like fold (d.58) includes 59 superfamilies with the most disparate activities. However, among these it is worth noting the existence of the heavy-metal-associated domain superfamily (HMA, d.58.17) that includes the protein Atx1, a eukaryotic copper chaperone with which PA4063 shares the peculiar surface-charge distribution shown in Fig. 4 ▸ (Banci *et al.*, 2006 ▸). In addition, the superfamily comprises the regulatory metal-binding domains of two zinc-efflux transporter of Gram-negative bacteria, namely ZntA and CDF, which act as cytosolic sensors that block efflux in the case of low zinc concentration (Blindauer, 2015 ▸). Although intriguing, this similarity must be taken carefully since, apart from sharing a similar fold and zinc-binding properties, PA4063 differs in cell localization, affinity and mode of binding.

The presence of flexible His-rich loops, with a known propensity to loosely bind zinc, in addition to the exposure and the relatively low affinity of the binding sites, indicates high mobility of Zn^2+^ (Kochańczyk *et al.*, 2015 ▸). This suggests that ions intercepted by PA4063 could be rapidly transferred to other proteins with higher affinity, similar to the role played by ZnuA in the corresponding ABC transport system, pointing to a role for PA4063 as a periplasmic zinc chaperone. This hypothesis would be coherent with the putative role of PA4064 and PA4065 as permease and ATP-binding components of an additional ABC transporter.

However, a notable difference exists between PA4063 and other periplasmic zinc chaperones: ZnuA, ZinT and AztD have zinc-binding sites with an affinity in the nanomolar range (Castelli *et al.*, 2013 ▸; Ilari *et al.*, 2014 ▸; Neupane *et al.*, 2019 ▸), namely around 2–3 orders of magnitude higher than that of PA4063. Thus, it is reasonable to assume that PA4063 effectively binds zinc only when its concentration in the periplasm exceeds a critical value. Based on this assumption, an alternative hypothesis may arise: PA4063 could act as a sensor for high zinc concentration and be involved in negative feedback control of zinc transport, being able to give an immediate response, faster than the transcriptional control mediated by Zur, in the case of a sudden increase in free zinc levels. A similar regulatory role has previously been hypothesized for the His-rich region of ZnuA (Wei *et al.*, 2007 ▸), which is also characterized by a micromolar affinity for zinc. Indeed, it has been proposed that upon zinc saturation this region of the protein could inhibit the ZnuA-mediated zinc-transport process.

Both hypotheses presented above imply that PA4063 has the ability to selectively interact with other proteins involved in controlling the bacterial response to zinc deficiency. Future studies will have to clarify whether this partner (or partners) belongs to the same operon, such as the putative permease PA4065, or to other zinc-transport systems.

## Supplementary Material

PDB reference: PA4063 from *Pseudomonas aeruginosa*, 7ahw

PDB reference: complex with zinc, 7amx

PDB reference: complex with zinc, space group *P*6_5_, 7bgo

PDB reference: complex with gold(I) for phasing, 7aly

Supplementary Figures. DOI: 10.1107/S2059798321009608/nz5005sup1.pdf

## Figures and Tables

**Table 1:** Data-collection and structure-refinement statistics Values in parentheses are for the outer shell.

	Tetragonal, apo	Tetragonal, zinc-bound	Hexagonal, gold-bound	Hexagonal, zinc-bound
PDB code	7ahw	7amx	7aly	7bgo
Data collection and processing				
Diffraction source	11.2C, Elettra	ID30A-3, ESRF	11.2C, Elettra	11.2C, Elettra
Wavelength (Å)	1.000	0.9677	1.000	1.271
Detector	PILATUS 6M	EIGER2 X 4M	PILATUS 6M	PILATUS 6M
Total rotation range (°)	180	300	225	300
Space group	*P*4_1_2_1_2	*P*4_1_2_1_2	*P*6_5_	*P*6_5_
*a*, *b*, *c* (Å)	65.85, 65.85, 101.22	65.47, 65.47, 102.07	122.63, 122.63, 102.64	122.00, 122.00, 102.26
α, β, γ (°)	90, 90, 90	90, 90, 90	90, 90, 120	90, 90, 120
Resolution range (Å)	46.55–1.95 (2.00–1.95)	62.47–2.85 (3.00–2.85)	47.16–2.70 (2.83–2.70)	46.94–3.30 (3.57–3.30)
No. of unique reflections	16839 (1154)	5107 (718)	22698 (3206)	12599 (2650)
Completeness (%)	99.9 (99.1)	99.6 (99.8)	93.7 (99.5)	96.1 (99.8)
Multiplicity	11.7 (9.1)	20.5 (20.7)	12.6 (13.0)	17.7 (17.9)
〈*I*/σ(*I*)〉	17.3 (1.9)	12.5 (1.2)	23.1 (2.1)	18.6 (1.6)
Half-set correlation CC_1/2 _(%)	99.9 (61.2)	98.9 (51.8)	99.9 (76.7)	99.9 (67.7)
*R*_meas_	0.088 (1.304)	0.213 (3.257)	0.096 (1.492)	0.15 (2.618)
*R*_p.i.m._	0.035 (0.590)	0.047 (0.708)	0.027 (0.412)	0.035 (0.616)
Overall *B* factor from Wilson plot (Å^2^)	36.5	87.6	70.0	123.5
Structure refinement				
Resolution range (Å)	42.32–1.95 (2.00–1.95)	44.21–2.85 (2.92–2.85)	46.25–2.70 (2.77–2.70)	46.94–3.30 (3.39–3.30)
Completeness (%)	99.9 (99.1)	99.5 (99.7)	93.7 (99.5)	96.0 (100)
No. of reflections, working set	16788 (1138)	4813 (342)	22698 (1691)	12561 (926)
No. of reflections, test set	825 (64)	258 (12)	1086 (77)	552 (38)
Final *R*_cryst_	0.193 (0.263)	0.185 (0.269)	0.194 (0.305)	0.177 (0.371)
Final *R*_free_	0.220 (0.329)	0.237 (0.262)	0.237 (0.339)	0.211 (0.370)
No. of molecules in asymmetric unit	1	1	3	3
Protein residues [sequence range]	133 [20–93, 121–179]	141 [15–93, 119–179]	137, 135, 134 [*A*, 19–96, 121–179; *B*, 19–95, 122–179; *C*, 19–94, 122–179]	140, 143, 141 [*A*, 15–95, 121–179; *B*, 14–95, 119–179; *C*, 15–94, 119–179]
No. of non-H atoms	1052	1060	3060	3210
No. of ions	—	2 [Zn]	1 [Au]	6 [Zn]
No. of water molecules	50	—	14	6
R.m.s. deviations				
Bond lengths (Å)	0.0145	0.0169	0.0106	0.0106
Angles (°)	1.835	1.686	1.632	1.811
Average *B* factors (Å^2^)				
Protein	42.35	86.5	79.95	143.6
Ion	—	89.4	70.1	159.4
Water	39.75	—	68.4	125.6
Ramachandran plot				
Most favoured	128 [98.2%]	128 [94.1%]	387 [98.2%]	391 [94.9%]
Allowed	1 [0.8%]	8 [5.9%]	7 [1.8%]	18 [4.4%]

**Table 2:** Thermodynamic parameters for metal–apo PA4063 interaction as assayed by ITC

Ligand	No. of binding sites	*K*_d_ (µ*M*)	Δ*G* (kcal mol^−1^)	Δ*H* (kcal mol^−1^)	−*T*Δ*S* (kcal mol^−1^)
Zn^2+^	2.08 ± 0.29	1.91 ± 0.42	−7.83 ± 0.64	−6.71 ± 0.17	−1.11 ± 0.53
Co^2+ ^	1.85 ± 0.02	4.27 ± 0.37	−7.33 ± 0.19	−5.40 ± 0.05	−1.94 ± 0.13
