# Supplementary material for: Structure and metal-binding properties of PA4063, a novel player in periplasmic zinc trafficking by Pseudomonas aeruginosa
Source: Acta Crystallogr D Struct Biol. 2021 Oct 20;77(Pt 11):1401–10. doi: 10.1107/S2059798321009608 (PMC8561739; doi:10.1107/S2059798321009608)
Supplement: Supplementary file 1 [file d-77-01401-sup1.pdf]

STRUCTURAL
BIOLOGY

Volume 77 (2021)

Supporting information for article:

Structure and metal-binding properties of PA4063, a novel player in periplasmic zinc trafficking by *Pseudomonas aeruginosa*

Annarita Fiorillo, Andrea Battistoni, Serena Ammendola, Valerio Secli, Serena Rinaldo, Francesca Cutruzzolà, Nicola Demitri and Andrea Ilari

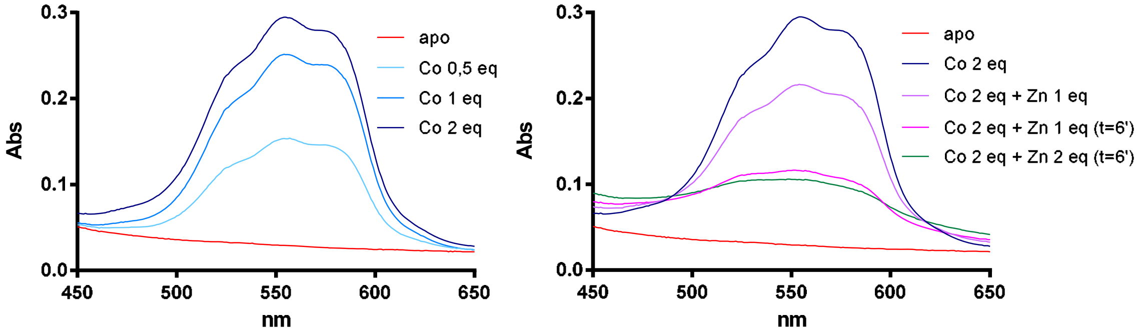

Figure S1 Visible absorption spectra of PA4063 in the 450-650 nm region. (a) Spectra were recorded after the addition of progressively higher quantities of cobalt to the apo-protein. Upon the addition of the metal a typical cobalt electronic absorption spectrum appeared, characterized by three overlapping peaks (a major peak at 557 nm and two shoulders around 530 and 580 nm), suggestive of a tetrahedral coordination geometry. As shown in Fig. S1*a*, by doubling the cobalt content from 0.5 to 1 equivalent, the absorption doubles proportionally. A further gradual addition of 0.5 equivalents, until reaching 2 equivalents, produces only a slight increase in absorption, while no change in absorbance was achieved by adding more than 2 equivalents of the metal (data not shown). After the addition of cobalt, no major differences were observed over time, suggesting that the conformation of the apo-protein is ready for metal binding and does not change significantly after cobalt incorporation. (b) To gain insight into the ability of PA4063 to bind zinc, we analysed the effect of zinc addition on the spectrum of the cobalt-bound protein. For this purpose, we carried out a gradual addition of ZnSO4 in aliquots of 0.5 zinc equivalents, as previously done with cobalt. The figure clearly shows that following the addition of zinc, there is a progressive time-dependent decrease in absorbance at 550 nm. The maximum reduction in absorbance at this wavelength was achieved after 6 min. This analysis indicates that zinc largely displaces cobalt from the protein, suggesting that PA4063 has a higher affinity for zinc than for cobalt.

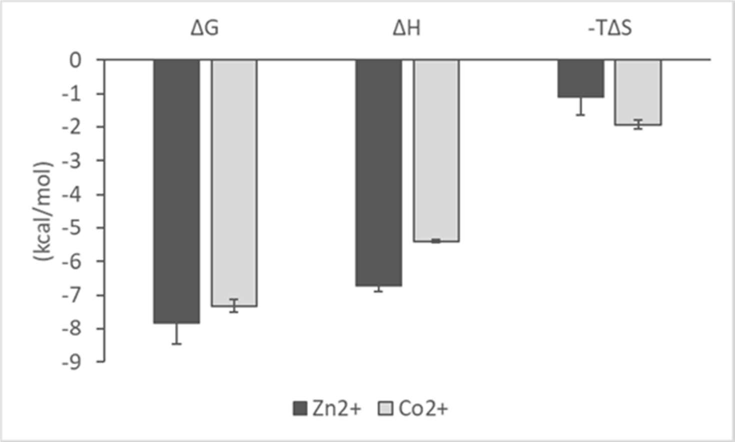

Figure S2 Plot of ΔG, ΔH, and −TΔS derived from the ITC data obtained from metal binding to PA4063. The thermodynamic signature indicates that binding of both metals is both enthalpy and entropy-driven.

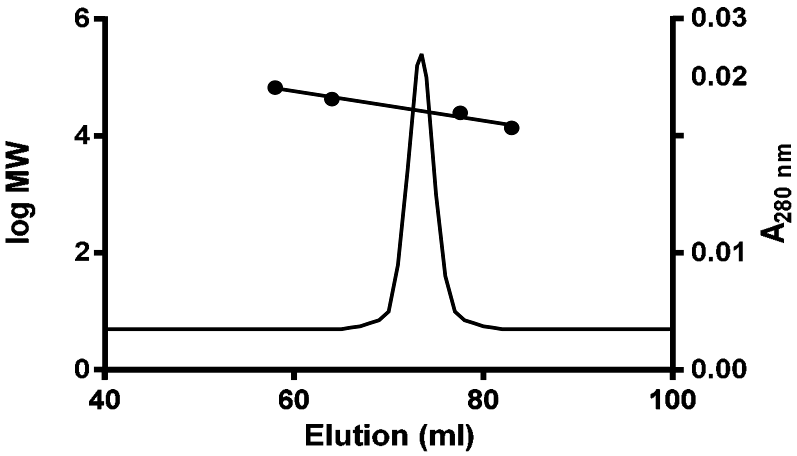

Figure S3 Elution profile of purified PA4063 injected onto a HiLoadTM 16/60 SuperdexTM 75 gel filtration FPLC column. The sample, at a concentration of 1 mg/ml, was eluted in 150 mM NaCl, 20 mM Tris-HCl, pH 7.5 at a rate of 1 ml/min. The column was calibrated with the following molecular weight markers (indicated by black circles): bovine serum albumin (67 kDa), ovoalbumin (43 kDa), chymotrypsinogen A (25 kDa) and ribonuclease A (13 kDa). The protein eluted as a single peak independently of the concentration and of the addition of EDTA or of Zn, with an apparent molecular weight of 26 kDa, slightly larger than the expected one (19,5 kDa). This increase in the hydrodynamic volume of the protein is coherent with the presence of unstructured regions, as revealed by diffraction experiments.

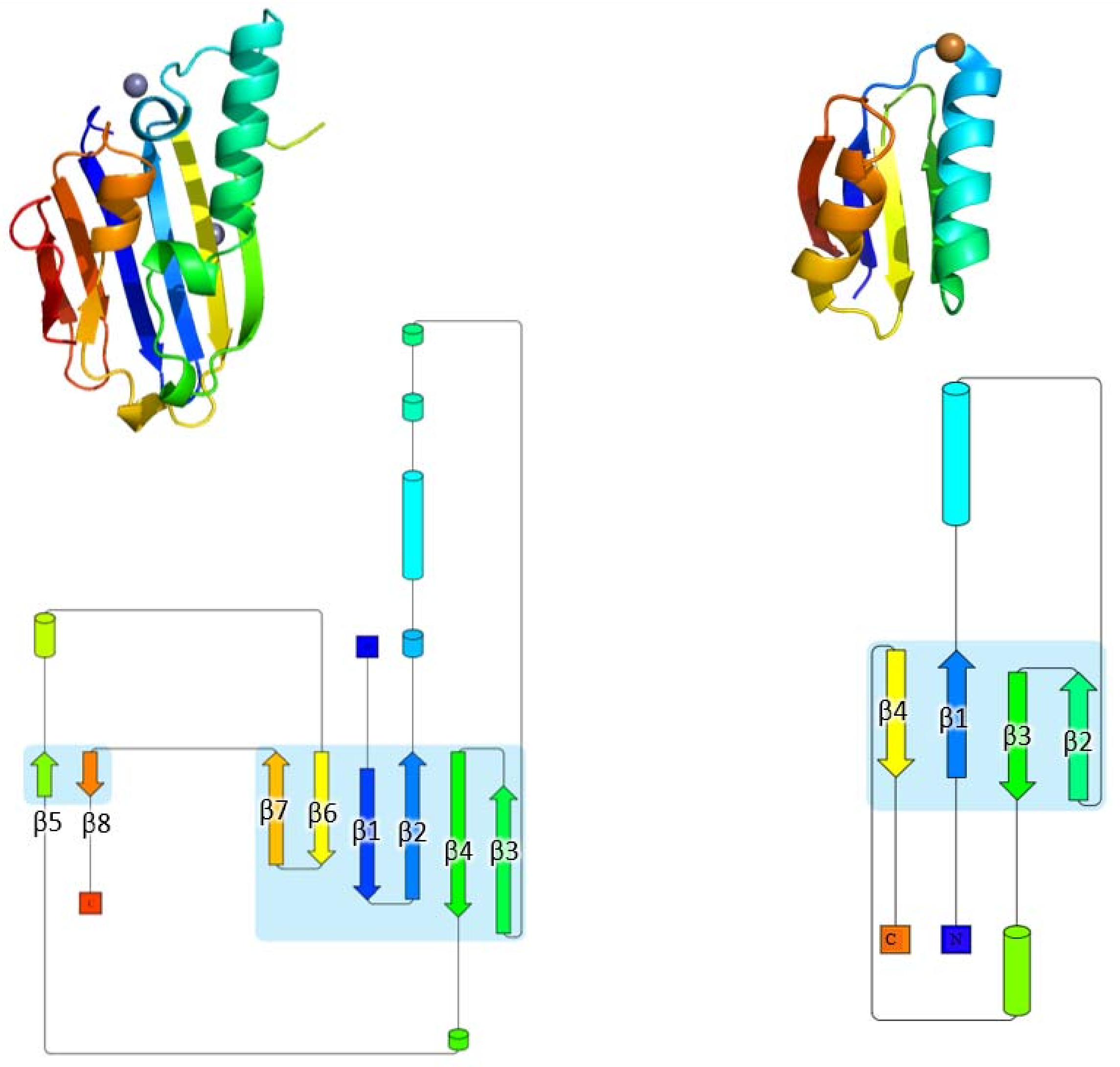

Figure S4 Topology diagrams for PA4063 (left) and Atox1 (right, pdb: 1fee) representative of canonical ferredoxin fold. Diagrams were generated by Pro-origami (A. Stivala, M. Wybrow, A. Wirth, J. Whisstock and P. Stuckey 2011 Automatic generation of protein structure cartoons with Pro-origami Bioinformatics 27(23):3315-3316 doi:10.1093/bioinformatics/btr575).

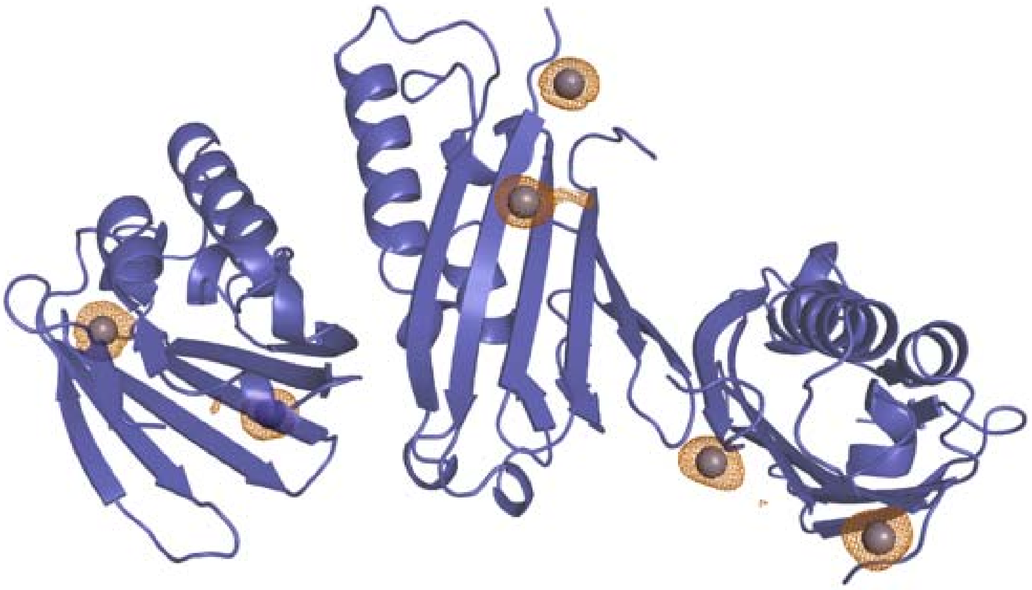

Figure S5 The figure represents the asymmetric unit (AU) of the hexagonal crystal form of PA4063 soaked in $Zn(CH_3CO_2)_2$. The anomalous difference density map (orange mesh), collected on the zinc absorption peak and here contoured at 4 σ, is shown for the whole AU, revealing two fully-occupied zinc binding sites for each monomer.

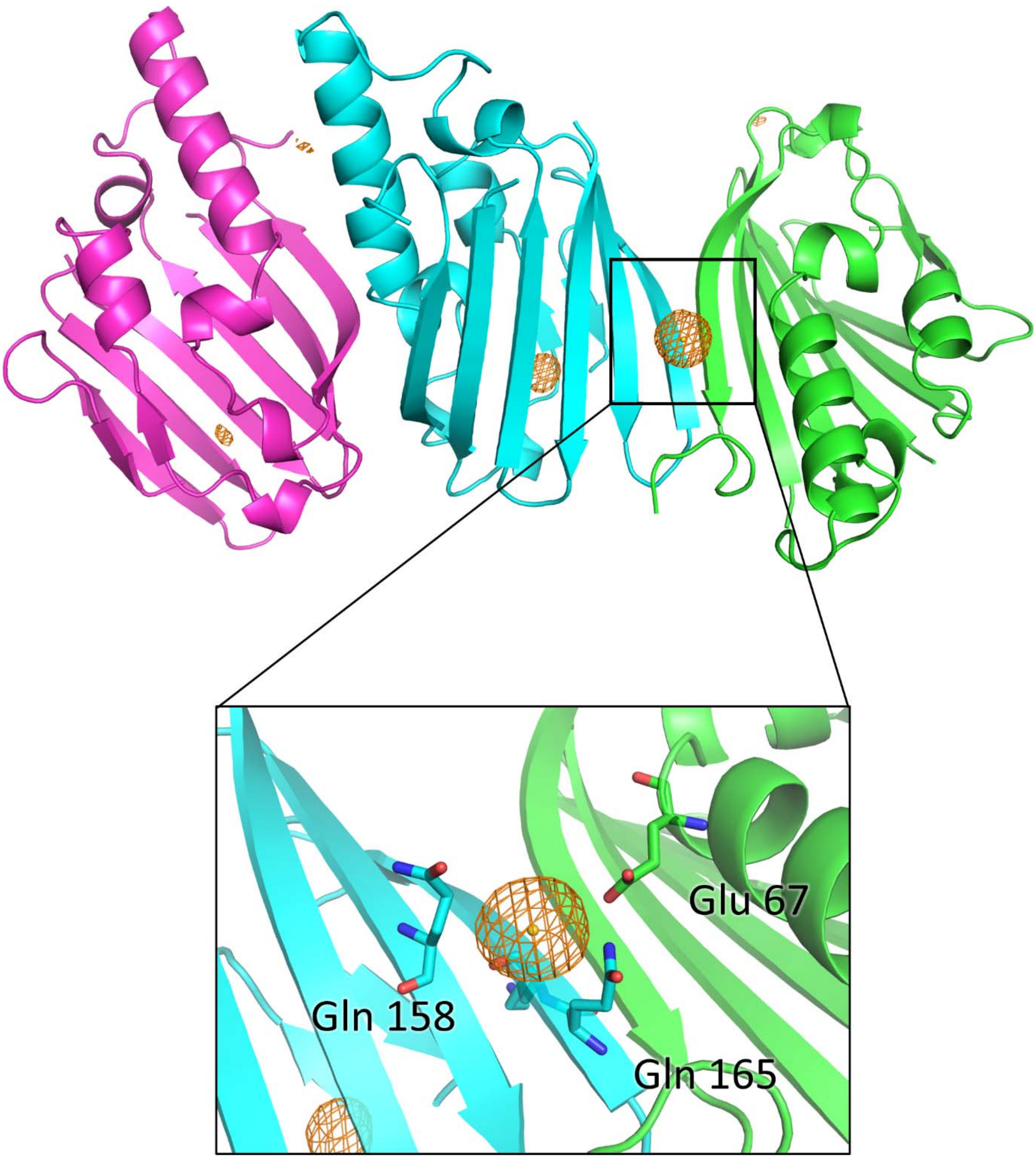

Figure S6 The figure represents the asymmetric unit (AU) of the hexagonal crystal form of PA4063 soaked in $KAu(CN)_2$. The anomalous difference density map (orange mesh), collected on the gold absorption peak and here contoured at 5 σ, is shown for the whole AU. The frame shows a detail of the residues surrounding the Au ion that has been included in the final model.

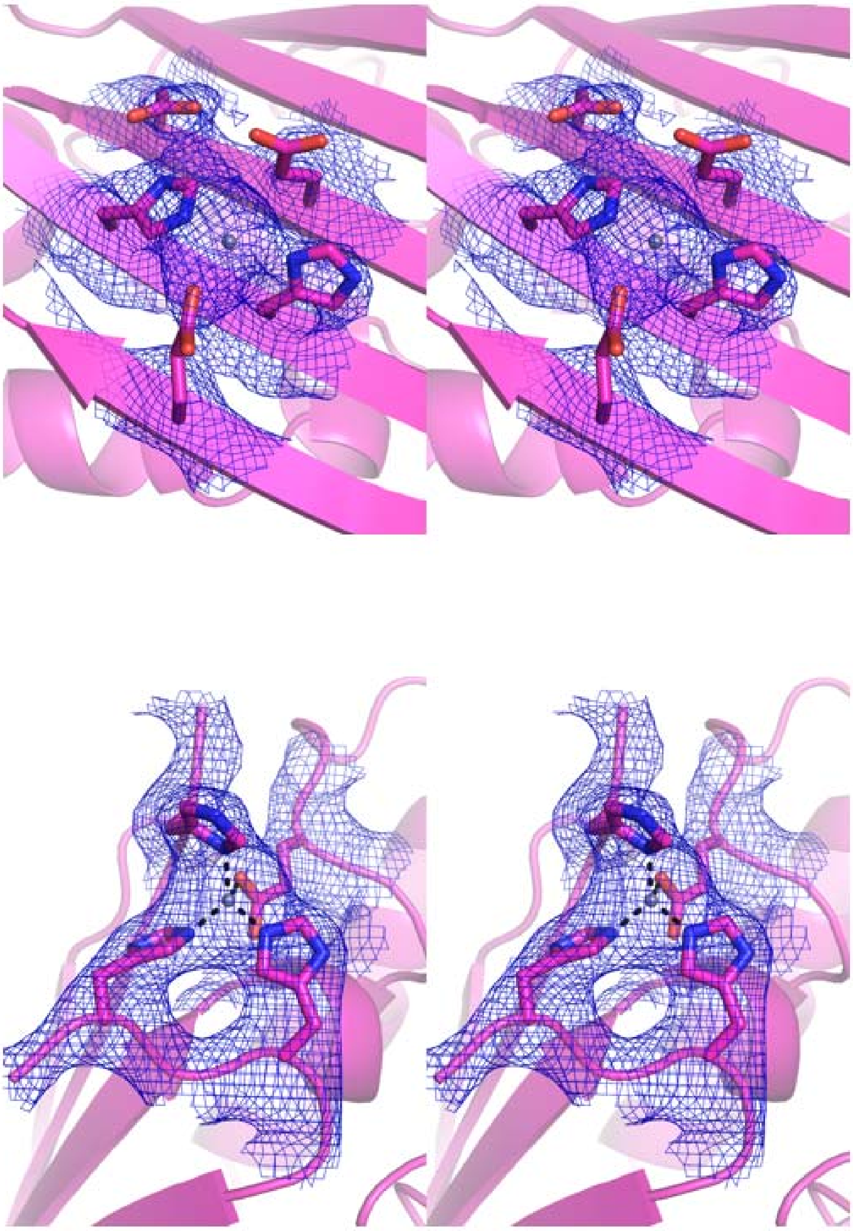

(*a*)

(*b*)

Figure S7 Stereo view of the of the zinc binding sites of tetragonal crystal. The electron density map (2mFo – DFc) is contoured at 1σ.
